# Cooperative Functions of ZnT1, Metallothionein and ZnT4 in the Cytoplasm Are Required for Full Activation of TNAP in the Early Secretory Pathway

**DOI:** 10.1371/journal.pone.0077445

**Published:** 2013-10-18

**Authors:** Shigeyuki Fujimoto, Naoya Itsumura, Tokuji Tsuji, Yasumi Anan, Natsuko Tsuji, Yasumitsu Ogra, Tomoki Kimura, Yusaku Miyamae, Seiji Masuda, Masaya Nagao, Taiho Kambe

**Affiliations:** 1 Division of Integrated Life Science, Graduate School of Biostudies, Kyoto University, Kyoto, Japan; 2 Laboratory of Chemical Toxicology and Environmental Health, Showa Pharmaceutical University, Machida, Tokyo, Japan; 3 High Technology Research Center, Showa Pharmaceutical University, Machida, Tokyo, Japan; 4 Department of Toxicology, Faculty of Pharmaceutical Sciences, Setsunan University, Hirakata, Osaka, Japan; University G. D'Annunzio, Italy

## Abstract

The activation process of secretory or membrane-bound zinc enzymes is thought to be a highly coordinated process involving zinc transport, trafficking, transfer and coordination. We have previously shown that secretory and membrane-bound zinc enzymes are activated in the early secretory pathway (ESP) via zinc-loading by the zinc transporter 5 (ZnT5)-ZnT6 hetero-complex and ZnT7 homo-complex (zinc transport complexes). However, how other proteins conducting zinc metabolism affect the activation of these enzymes remains unknown. Here, we investigated this issue by disruption and re-expression of genes known to be involved in cytoplasmic zinc metabolism, using a zinc enzyme, tissue non-specific alkaline phosphatase (TNAP), as a reporter. We found that TNAP activity was significantly reduced in cells deficient in *ZnT1*, *Metallothionein* (*MT*) and *ZnT4* genes (*ZnT1* ^−/−^ *MT* ^−/−^ *ZnT4* ^−/−^ cells), in spite of increased cytosolic zinc levels. The reduced TNAP activity in *ZnT1* ^−/−^ *MT* ^−/−^ *ZnT4* ^−/−^ cells was not restored when cytosolic zinc levels were normalized to levels comparable with those of wild-type cells, but was reversely restored by extreme zinc supplementation via zinc-loading by the zinc transport complexes. Moreover, the reduced TNAP activity was adequately restored by re-expression of mammalian counterparts of ZnT1, MT and ZnT4, but not by zinc transport-incompetent mutants of ZnT1 and ZnT4. In *ZnT1* ^−/−^ *MT* ^−/−^ *ZnT4* ^−/−^ cells, the secretory pathway normally operates. These findings suggest that cooperative zinc handling of ZnT1, MT and ZnT4 in the cytoplasm is required for full activation of TNAP in the ESP, and present clear evidence that the activation process of zinc enzymes is elaborately controlled.

## Introduction

Of all transition metals, zinc is the most widely used catalytic and structural factor in proteins [1], [2]. Zinc proteomics predicts that approximately 10% of proteins encoded in the human genome have a motif that potentially binds to zinc [3], [4]. Among these proteins, approximately 1000 are enzymes, which are involved in diverse physiological functions and can be classified into six major classes [5]. Most zinc enzymes use zinc as a catalytic component [4], and therefore zinc coordination (metalation) following zinc transport, trafficking and transfer is fundamental for enzyme activity. The molecular mechanism for this activation process, however, remains unclear. Metallothionein (MT) has been suggested to control the activation of cytoplasmic zinc enzymes [6] and to play a zinc chaperoning role in *in vitro* studies [2], [7]. All zinc transport proteins, including zinc transporters (ZnTs) and ZRT/IRT-related proteins (ZIPs), would potentially be involved in enzyme activation via zinc transport across the cell membrane [8], [9]. However, at present there is little direct evidence.

Secretory and membrane-bound zinc enzymes, such as matrix metalloproteinases, angiotensin-converting enzymes [10], A disintegrin and metalloproteinase (ADAM) family proteins [11], and alkaline phosphatase [12], are thought to become functional by incorporating zinc in the early secretory pathway (ESP) before reaching their final destination. Thus, zinc transport into the lumen of the ESP is one of the crucial steps for enzyme activation [9]. Compared with the well-known activation process of secretory cuproenzymes by Atox1-ATP7A/ATP7B pathways [13]–[15], understanding of the activation process of secretory and membrane-bound zinc enzymes has been less clear. We have previously shown that the ZnT5-ZnT6 hetero-complex and ZnT7 homo-complex (zinc transport complexes) are employed as zinc entry routes into the ESP [16], [17]. We have also shown that the zinc transport complexes are indispensable for the activation of secretory and membrane-bound zinc enzymes by converting them from the apo to the holo form using tissue non-specific alkaline phosphatase (TNAP) as a reporter enzyme [18]. However, how other proteins involved in cellular zinc metabolism affect this activation process remains unknown [19].

Here, we examined the TNAP activation process by establishing a series of cells deficient in genes encoding molecules known to be involved in cytoplasmic zinc metabolism. Specifically, we disrupted the *ZnT1*, *MT* and *ZnT4* genes in the cells, whose products play pivotal roles in the maintenance of cellular zinc homeostasis [8], [15], [20] via regulatory mechanisms called ‘zinc buffering’ and ‘muffling’ [21], [22]. Using these deficient cells, we show that ZnT1, MT and ZnT4 contribute to full activation of TNAP in the ESP, upstream of the zinc transport complexes.

## Materials and Methods

### Cell culture and transient transfection

Chicken B lymphocyte-derived DT40 cells were maintained in RPMI 1640 (Nacalai Tesque, Kyoto, Japan) supplemented with 10% (v/v) heat-inactivated fetal calf serum (FCS; Multiser, Trace Scientific, Melbourne, Australia), 1% (v/v) chicken serum (Invitrogen, Carlsbad, CA, USA) and 50 µM 2-mercaptoethanol (Sigma, St. Louis, MO, USA) at 39.5°C as previously described [23]. Zinc-deficient medium was prepared using fetal calf and chicken serum treated with Chelex-100 resin as described previously [24]. To evaluate cell viability against extracellular high zinc, the cells were cultured in the presence of 50–80 µM ZnSO_4_ for 72 h. The numbers of viable cells, judged by exclusion of trypan blue, were then counted and relative viability was determined as previously described [25]. For transient transfection, circular plasmids (20 µg) were electroporated into cells (5×10^6^ cells) as described previously [26].

### Plasmid construction

The ∼12-kb chicken *MT* (c*MT*) genes were amplified with gene-specific primers by KOD-FX polymerase (TOYOBO, Osaka, Japan) using DT40 genomic DNA as a template. The long or short arm was PCR-amplified and subcloned downstream or upstream of the drug selection marker cassettes, including drug-resistant genes (*Bsr* or *HisD*) flanked by mutant loxP sites. These targeting vectors were designed to disrupt both c*MT1* and c*MT2*, which are ∼2 kb apart (Fig. S1). *MT1* and *MT2* were assigned as described elsewhere [27]. Plasmids to express epitope-tagged human ZnT1 (hZnT1), hZnT2, hZnT4, hZnT5, hZnT6, hZnT7 and mouse Mt-I (mMt-I) were constructed by inserting each cDNA into pA-Puro, pA-Zeocin, pA-Ecogpt or pA-Neo vectors [18]. Introduction of mutation into hZnT1 or hZnT4 cDNA was carried out by the two-step PCR method, and amplified cDNAs were sequenced in both directions. All plasmids were linearized with appropriate restriction enzymes prior to electroporation for establishing the stable transfectant. To construct the secretory Cypridina luciferase expression plasmid for transient transfection study, chicken β-actin promoter was inserted into the multiple cloning site of pMCS-*Cypridina* Luc (Thermo Scientific, Waltham, MA, USA). Construction of MT-I-Luc was as previously described [28].

### Generation of mutant cells and stable transfectants

The experimental strategy and targeting vectors used are shown in Fig. S1. *ZnT1* ^−/−^ and *ZnT4* ^−/−^ were established as described previously [18]. To obtain *MT* ^−/−^ cells, wild-type (WT) DT40 cells were transfected sequentially with c*MT*-*Bsr* and c*MT*-*HisD* targeting constructs. To generate *ZnT1* ^−/−^ *MT* ^−/−^ and *ZnT1* ^−/−^ *MT* ^−/−^ *ZnT4* ^−/−^ cells, the drug selection marker cassettes in *ZnT1* ^−/−^ cells or *ZnT1* ^−/−^ *MT* ^−/−^ cells were excised according to methods described previously [16]. Briefly, these cells stably harboring pANMerCreMer plasmid were cultured for 2 days in the presence of 200 nM 4-hydroxytamoxifen (Sigma), which translocates MerCreMer protein (estrogen receptor-Cre recombinase fusion protein) into the nucleus, thereby recombining DNA at mutant loxP sites. Excision of the drug selection marker cassettes was confirmed by loss of drug resistance. The established cells were transfected sequentially with other targeting vectors described in Fig. S1.

### Measurement of TNAP activity, luciferase activities and determination of zinc contents

TNAP activity was measured as previously described [17]. In this study, calf intestine alkaline phosphatase (Promega, Madison, WI, USA) was used to make a standard curve. Therefore, units of TNAP activity were altered to be approximately 50-fold lower than those in our previous study [23]. The activities of firefly and renilla luciferase were measured using a dual-luciferase reporter assay system (Promega) as described previously [26]. Firefly luciferase activity was divided by renilla luciferase activity for normalization of transfection efficiency. The activity of secretory *Cypridina* luciferase in the spent medium was measured using Pierce *Cypridina* Luciferase Glow Assay Kit (Thermo Scientific). Zinc content in cells was determined using an inductive coupled plasma mass spectrometer (Agilent7500ce, Agilent Technologies, Hachioji, Japan), as described previously [29].

### RT-PCR

Total RNA was isolated from harvested cells using Sepasol I (Nacalai Tesque). Reverse transcription was performed using a ReverTra Ace (TOYOBO), and PCR was performed using KOD-FX (TOYOBO). Information on PCR primers and conditions used are listed in Table S1.

### Immunoblotting

Immunoblotting was performed as described previously [23]. The following antibodies were used: anti-FLAG M2 (Sigma; 1∶2000 dilution), anti-FLAG tag antibody (anti-DDDDK; MBL, Nagoya, Japan; 1∶3000), anti-HA HA-11 (COVANCE, Emeryville, CA, USA; 1∶3000), anti-MT (Dako, Glostrup, Denmark; 1∶3000), anti-tubulin (Sigma; 1∶10000) and anti-calnexin (Stressgen, Ann Arbor, MI, USA; 1∶2000). For detection of MT, some modifications were performed as described elsewhere [30]. Immobilon Western Chemiluminescent HRP Substrates (Millipore, Billerica, MA, USA) or SuperSignal West Femto Maximum Sensitivity Substrate (Pierce, Rockford, IL, USA) were used for detection. The fluoroimage was obtained using a LAS1000 plus image analyzer (Fujifilm, Tokyo, Japan).

### Immunofluorescence staining

Immunostaining for FLAG-hZnT1 and hZnT4-HA expressed in the cells was performed as previously described [18]. Briefly, the cells were stained with anti-HA antibody (1∶3000; COVANCE) or anti-FLAG tag antibody (anti-DDDDK; 1∶3000; MBL) followed by Alexa 488-conjugated goat anti-rabbit IgG (1∶3000; Molecular Probes, Eugene, OR, USA), or followed by Alexa 594-conjugated goat anti-mouse IgG (1∶3000; Molecular Probes). Immunostaining for the surface IgM was performed with anti-chicken IgM antibody M4 (1∶400; Beckman Instruments, Inc., Fullerton, CA, USA), followed by Alexa 594-conjugated goat anti-mouse IgG (1∶400; Molecular Probes). The stained cells were observed under a fluorescent microscope (Olympus, Tokyo, Japan). Images were analyzed using Adobe Photoshop Elements (Adobe Systems, Inc., San Jose, CA, USA).

### Cell surface biotinylation

DT40 cells cultured in the fresh normal medium for 24 h were washed twice with ice-cold phosphate buffered saline and then EZ-Link, a Sulfo-NHS-SS-Biotin reagent (Pierce) was added to biotinylated lysine residues exposed on the extracellular surface of the plasma membrane. Biotinylated proteins were recovered from the streptavidin-coupled beads in 6X SDS sample buffer and then subjected to lectin blotting. Biotinylated proteins were detected using the Streptavidin Biotin Complex Peroxidase Kit (Nacalai Tesque). Lectin blotting was performed using biotin-wheat germ agglutinin (WGA, Seikagaku Kogyo, Tokyo, Japan) as described previously [26].

### Statistical analyses

All data are depicted as mean ± SD. Statistical significance was determined by Student's t test and accepted at p<0.01.

## Results

### Establishment and characterization of DT40 cells deficient in *cZnT1, cMT* and *cZnT4* genes

To investigate how proteins conducting cytoplasmic zinc metabolism affect TNAP activation, we established DT40 cells deficient in a series of chicken *ZnT1* (c*ZnT1*), c*MT* and/or c*ZnT4* genes (*ZnT1* ^−/−^, *MT* ^−/−^, *ZnT4* ^−/−^, *ZnT1* ^−/−^ *MT* ^−/−^ and *ZnT1* ^−/−^ *MT* ^−/−^ *ZnT4* ^−/−^ cells) (Fig. S1). Because targeting vectors for the c*MT* gene were designed to disrupt both c*MT1* and c*MT2* genes simultaneously, the *MT* ^−/−^ cells established do not express any MTs. DT40 cells do not express a functional transcript of c*ZnT2* [18], although ZnT2 is known to contribute to cytoplasmic zinc homeostasis [9], [19], [20], which precluded establishing *ZnT2* ^−/−^ cells in this study.

**Figure 1:**
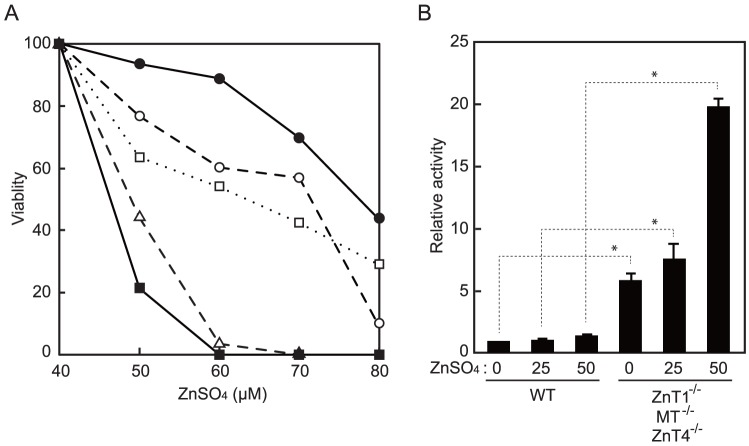
Cytosolic zinc levels are increased in *ZnT1* ^−/−^ *MT* ^−/−^ *ZnT4* ^−/−^ cells. (**A**) Zinc sensitivity of DT40 cells deficient in c*ZnT1*, c*MT* and/or c*ZnT4* genes. Cells were grown in the presence of the indicated concentrations of ZnSO_4_ for 72 h and the number of living cells was counted (plotted as a percentage of living cells at 40 µM ZnSO_4_ for each group of cells). •, wild-type (WT); ○, *MT* ^−/−^; □, *ZnT1* ^−/−^; △, *ZnT1* ^−/−^ *MT* ^−/−^; ▪, *ZnT1* ^−/−^ *MT* ^−/−^ *ZnT4* ^−/−^ cells. Each value is the mean of two independent experiments. (**B**) Effects of zinc on MT-I luciferase reporter expression in WT cells and *ZnT1* ^−/−^ *MT* ^−/−^ *ZnT4* ^−/−^ cells. Both cell types were transiently transfected and cultured in the presence of 0, 25 or 50 µM ZnSO_4_ for 12 h. Relative activity of luciferase is shown (the luciferase activity of WT cells cultured without ZnSO_4_ is defined as 1). Each value is the mean ± SD of three independent experiments (*P<0.01).

Consistent with previous literature on zinc-sensitive mutant baby hamster kidney cell lines that neither express endogenous MTs nor have functional ZnT1 [31], *ZnT1* ^−/−^ *MT* ^−/−^ cells showed a zinc-sensitive phenotype to extracellular high zinc (Fig. 1A). *ZnT1* ^−/−^ *MT* ^−/−^ *ZnT4* ^−/−^ cells were more sensitive to high zinc, and did not grow in the presence of 60 µM or more ZnSO_4_ (Fig. 1A). An additional study using the MT-I luciferase reporter, which is widely used to assess cytosolic zinc levels, indicated increased luciferase activity in *ZnT1* ^−/−^ *MT* ^−/−^ *ZnT4* ^−/−^ cells compared with WT cells (Fig. 1B). These results confirmed that *ZnT1* ^−/−^ *MT* ^−/−^ *ZnT4* ^−/−^ cells have increased cytosolic zinc levels compared with WT cells. The zinc-sensitive phenotype of *ZnT1* ^−/−^ *MT* ^−/−^ *ZnT4* ^−/−^ cells was reversed by re-expressing hZnT1, mMt-I, or hZnT4, although the relative contribution of hZnT4 was moderate (Table 1). The effects of re-expression were also confirmed using the MT-I luciferase reporter assay (data not shown). Taken together, loss of *ZnT1*, *MT* and *ZnT4* genes causes cytosolic zinc levels to increase in DT40 cells.

**Table 1:** Restoration of resistance of *ZnT1* ^−/−^ *MT* ^−/−^ *ZnT4* ^−/−^ cells by expression of the indicated genes against high zinc toxicity.

	ZnSO_4_ (µM)			
Genes	50	60	70	80
-	++	-	-	-
FLAG-hZnT1	++	++	++	++
mMt-I	++	++	++	++
hZnT4-HA	++	++	+	-
hZnT2-HA	++	++	++	++
FLAG-hZnT1 H43A	++	-	-	-
hZnT4-HA H146A	++	-	-	-

The relative contribution of hZnT1, mMt-I or hZnT4 re-expression in *ZnT1* ^−/−^ *MT* ^−/−^ *ZnT4* ^−/−^ cells against zinc toxicity was determined by counting the number of cells after 72 h exposure to the indicated concentrations of ZnSO_4_. The relative contribution of exogenous expression of hZnT2 or mutant hZnT1 or hZnT4 is also indicated. Relative values presented are evaluations of the averages of three independent experiments. ++: growing to confluence; +: less growth compared with ++ (20–50% relative to ++); -: not growing.

### ZnT1, ZnT4 and MT are all involved in the activation process of TNAP

**Figure 2:**
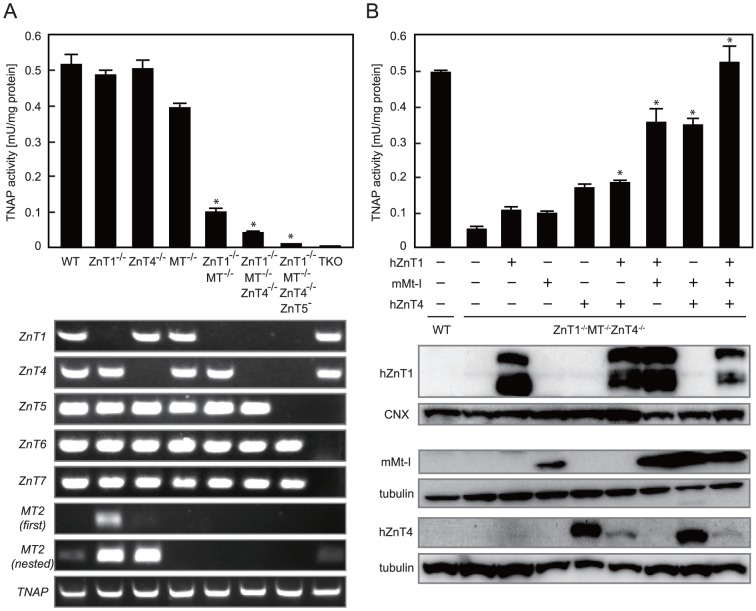
ZnT1, MT and ZnT4 are all involved in the activation process of TNAP. (**A**) TNAP activity decreased in DT40 cells deficient in genes coding for c*ZnT1*, c*MT* and/or c*ZnT4* (*upper panel*). TNAP activity of total cellular protein prepared from the indicated cells is expressed as the mean ± SD of three independent experiments (*P<0.01 vs. WT cells). Disruption of each gene was confirmed by RT-PCR using the appropriate primers (*lower panel*). *cMT2* expression is shown in duplicate (first and nested) to show significant or moderate induction of *cMT2* mRNA expression in *ZnT1* ^−/−^ or *ZnT4* ^−/−^ cells. Confirmation of c*TNAP* expression is also shown. TKO; *ZnT5* ^−^ *ZnT6* ^−/−^ *ZnT7* ^−/−^ cells. (**B**) Re-expression of hZnT1, mMt-I and/or hZnT4 restored TNAP activity in *ZnT1* ^−/−^ *MT* ^−/−^ *ZnT4* ^−/−^ cells (*upper panel*). Expression of FLAG-hZnT1, mMt-I and hZnT4-HA was confirmed by immunoblot analysis using total cellular or membrane proteins prepared from the indicated cells. Tubulin and calnexin (CNX) are shown as loading controls (*lower panels*). TNAP activity is expressed as the mean ± SD of three independent experiments (*P<0.01 vs. *ZnT1* ^−/−^ *MT* ^−/−^ *ZnT4* ^−/−^ cells).

TNAP activity was measured in *ZnT1* ^−/−^ *MT* ^−/−^ *ZnT4* ^−/−^ cells and other knockout cells established in this study. We first assumed that zinc-dependent TNAP activity may be enhanced in these cells because of the increase in cytosolic zinc levels. TNAP activity in *ZnT1* ^−/−^ and ZnT4^−/−^ cells remained unchanged, but the activity in *MT* ^−/−^ cells decreased slightly (Fig. 2A). Contrary to our assumption, TNAP activity significantly decreased in *ZnT1* ^−/−^ *MT* ^−/−^ cells, while that in *ZnT1* ^−/−^ *MT* ^−/−^ *ZnT4* ^−/−^ cells decreased by approximately 90% when compared with that in WT cells. *TNAP* mRNA expression levels were almost the same among these cells (Fig. 2A).

We then confirmed that the decrease in TNAP activity was attributed to the loss of ZnT1, MT and ZnT4. Single re-expression of the human or mouse counterparts of these genes, specifically hZnT1, mMt-I or hZnT4, in *ZnT1* ^−/−^ *MT* ^−/−^ *ZnT4* ^−/−^ cells only slightly restored the reduced TNAP activity, but re-expression of at least two of the counterparts moderately restored activity. Re-expression of all counterparts restored reduced TNAP activity to that of WT cells (Fig. 2B). These results indicate that ZnT1, MT and ZnT4 are all required for maintaining maximal activity of TNAP.

### The zinc transport complexes cannot fully operate for TNAP activation in *ZnT1* ^−/−^ *MT* ^−/−^ *ZnT4* ^−/−^ cells

**Figure 3:**
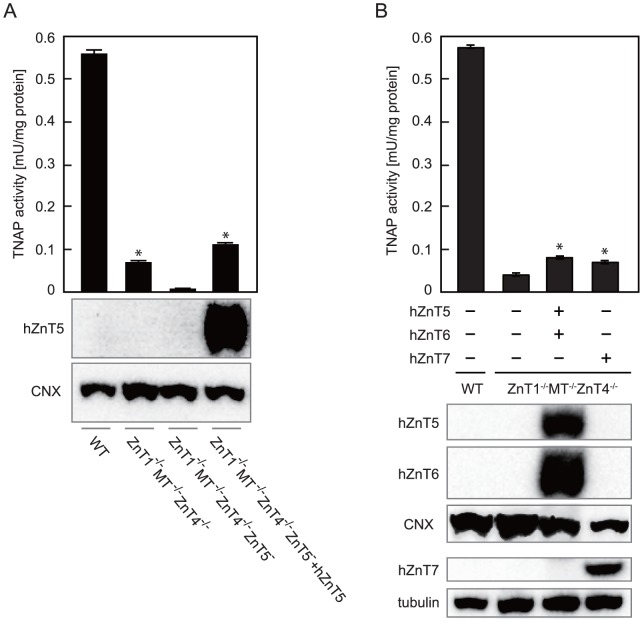
The zinc transport complexes cannot fully operate for TNAP activation in *ZnT1* ^−/−^ *MT* ^−/−^ *ZnT4* ^−/−^ cells. (**A**) The residual TNAP activity in *ZnT1* ^−/−^ *MT* ^−/−^ *ZnT4* ^−/−^ cells was dependent on zinc-loading by the ZnT5-ZnT6 hetero-complex. TNAP activity of the total cellular protein prepared from the indicated cells is expressed as the mean ± SD of three independent experiments (*P<0.01 vs. *ZnT1* ^−/−^ *MT* ^−/−^ *ZnT4* ^−/−^ *ZnT5* ^−/−^ cells, *upper panel*). Expression of FLAG-hZnT5 in *ZnT1* ^−/−^ *MT* ^−/−^ *ZnT4* ^−/−^ *ZnT5* ^−^ cells was confirmed by immunoblot analysis using membrane proteins. Calnexin (CNX) is shown as a loading control (*lower panels*). (**B**) Over-expression of hZnT5 and hZnT6 or hZnT7 failed to restore the reduced TNAP activity in *ZnT1* ^−/−^ *MT* ^−/−^ *ZnT4* ^−/−^ cells. TNAP activity of the total cellular protein prepared from the indicated cells is expressed as the mean ± SD of three independent experiments (*P<0.01 vs. WT cells, *upper panel*). Expression of FLAG-hZnT5 and HA-hZnT6 or HA-hZnT7 in *ZnT1* ^−/−^ *MT* ^−/−^ *ZnT4* ^−/−^ cells was confirmed by immunoblot analysis as in A (*lower panels*).

We examined whether the zinc transport complexes, which are indispensable for the activation of TNAP [9], can transport zinc into the ESP to activate TNAP in *ZnT1* ^−/−^ *MT* ^−/−^ *ZnT4* ^−/−^ cells. Disruption of the c*ZnT5* gene in *ZnT1* ^−/−^ *MT* ^−/−^ *ZnT4* ^−/−^ cells markedly reduced TNAP activity to a level similar to that of *ZnT5* ^−^ *ZnT6* ^−/−^ *ZnT7* ^−/−^ cells (termed TKO cells in our previous studies [16], [17]) (Fig. 2A and Fig. 3A, lane 3). Re-expression of hZnT5 in *ZnT1* ^−/−^ *MT* ^−/−^ *ZnT4* ^−/−^ *ZnT5* ^−^ cells restored TNAP activity to a level comparable to that in *ZnT1* ^−/−^ *MT* ^−/−^ *ZnT4* ^−/−^ cells (Fig. 3A, lane 4), suggesting that residual TNAP activity was caused via zinc transport into the ESP by the zinc transport complexes. Moreover, we established *ZnT1* ^−/−^ *MT* ^−/−^ *ZnT4* ^−/−^ cells stably over-expressing hZnT5 and hZnT6 simultaneously or over-expressing hZnT7. However, TNAP activity was not significantly enhanced and could not be restored to levels comparable to that of WT cells in both cases (Fig. 3B), which was in sharp contrast to the results in TKO cells stably expressing hZnT5/hZnT6 or hZnT7, where similar levels of ZnTs expression fully restored reduced TNAP activity [16]–[18]. Thus, the zinc transport complexes cannot fully operate for TNAP activation in *ZnT1* ^−/−^ *MT* ^−/−^ *ZnT4* ^−/−^ cells.

### The increased cytosolic zinc levels in *ZnT1* ^−/−^ *MT* ^−/−^ *ZnT4* ^−/−^ cells is not responsible for reduced TNAP activity

**Figure 4:**
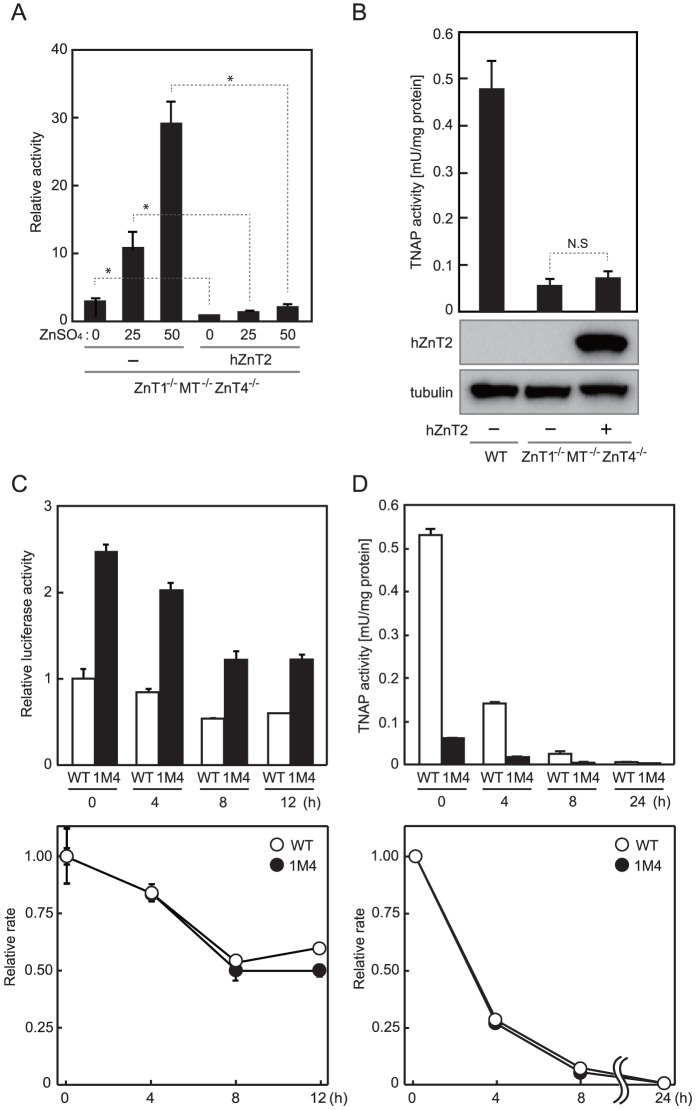
Reduction of the cytosolic zinc levels in ZnT1^−/−^MT^−/−^ZnT4^−/−^ cells did not restore reduced TNAP activity. (**A**) Exogenous expression of hZnT2 reduced the cytosolic zinc levels in *ZnT1* ^−/−^ *MT* ^−/−^ *ZnT4* ^−/−^ cells. *ZnT1* ^−/−^ *MT* ^−/−^ *ZnT4* ^−/−^ cells or *ZnT1* ^−/−^ *MT* ^−/−^ *ZnT4* ^−/−^ cells stably expressing hZnT2-HA were transiently transfected with MT-I luciferase as in Fig. 1B. Relative activity of luciferase is shown (the luciferase activity of *ZnT1* ^−/−^ *MT* ^−/−^ *ZnT4* ^−/−^ cells stably expressing hZnT2 cultured without ZnSO_4_ is defined as 1). Each value is the mean ± SD of three independent experiments (*P<0.01). (**B**) TNAP activity of the total cellular protein prepared from the indicated cells is expressed as the mean ± SD of three independent experiments (N.S., not significant, *upper panel*). Expression of hZnT2 was confirmed by immunoblot analysis using total cellular proteins. Tubulin is shown as a loading control (*lower panels*). (**C**) The change in cytosolic zinc levels in WT and *ZnT1* ^−/−^ *MT* ^−/−^ *ZnT4* ^−/−^ cells during zinc-deficient culture. Both cell lines were transiently transfected with MT-I luciferase, cultured in normal medium for 9 h, and then cultured in zinc-deficient medium for the indicated period of time prior to measuring luciferase activity. (**D**) The change in TNAP activity in WT and *ZnT1* ^−/−^ *MT* ^−/−^ *ZnT4* ^−/−^ cells during zinc-deficient culture. Both cells cultured in normal medium were washed once and then cultured in zinc-deficient medium for the indicated period. TNAP activity is expressed as the mean ± SD of three independent experiments. In the lower panels of *C* and *D*, relative rates of change are plotted with values of WT or *ZnT1* ^−/−^ *MT* ^−/−^ *ZnT4* ^−/−^ cells at time 0 h defined as 1. 1M4; *ZnT1* ^−/−^ *MT* ^−/−^ *ZnT4* ^−/−^ cells.

We considered two possibilities that caused reduced TNAP activity in *ZnT1* ^−/−^ *MT* ^−/−^ *ZnT4* ^−/−^ cells; one is that the increased cytosolic zinc levels may result in disturbance of cytosolic zinc metabolism and thus negatively affect TNAP activation by impairing the ability of the zinc transport complexes to efficiently activate TNAP, and the other is that zinc handling by ZnT1, MT and ZnT4 may be important for TNAP activation via zinc-loading by the zinc transport complexes independently of the cytosolic zinc levels. To explore the possibilities, we decreased the cytosolic zinc levels in *ZnT1* ^−/−^ *MT* ^−/−^ *ZnT4* ^−/−^ cells by exogenously expressing hZnT2, and measured TNAP activity, because ZnT2 is known to have such activity by mobilizing zinc into the vesicles where ZnT2 is localized [25], [32], [33]. As expected, expression of hZnT2 made the cells resistant to high zinc toxicity (Table 1), and decreased the cytosolic zinc levels in *ZnT1* ^−/−^ *MT* ^−/−^ *ZnT4* ^−/−^ cells, as shown by MT-I luciferase reporter assays (Fig. 4A). The reduced TNAP activity, however, was not significantly changed by expression of hZnT2 in *ZnT1* ^−/−^ *MT* ^−/−^ *ZnT4* ^−/−^ cells (Fig. 4B), suggesting that the increased cytosolic zinc levels in *ZnT1* ^−/−^ *MT* ^−/−^ *ZnT4* ^−/−^ cells is independent of the reduction of TNAP activation. We also examined the effects of increased cytosolic zinc levels in *ZnT1* ^−/−^ *MT* ^−/−^ *ZnT4* ^−/−^ cells on TNAP activation by periodically measuring TNAP activity in zinc-deficient cultures. The MT-I luciferase reporter assay showed that zinc levels in *ZnT1* ^−/−^ *MT* ^−/−^ *ZnT4* ^−/−^ cells cultured in zinc-deficient conditions decreased to similar levels to that of WT cells cultured in normal medium (time 0 h) (Fig. 4C, *upper panel*). However, TNAP activity did not increase, but promptly decreased during the culture (Fig. 4D, *upper panel*). These results strongly suggest that it is unlikely that cytosolic zinc levels in *ZnT1* ^−/−^ *MT* ^−/−^ *ZnT4* ^−/−^ cells are crucial for TNAP activation.

While cytosolic zinc levels in *ZnT1* ^−/−^ *MT* ^−/−^ *ZnT4* ^−/−^ cells were enhanced compared with those in WT cells, the relative rate of decrease in zinc levels during zinc-deficient culture was almost the same between WT and *ZnT1* ^−/−^ *MT* ^−/−^ *ZnT4* ^−/−^ cells (Fig. 4C, *lower panel*). Similarly, the relative rate of decrease in TNAP activity was almost the same between in both cells (Fig. 4D, *lower panel*), although the absolute activities were markedly different. In addition, no significant differences were found in the rates of change in cellular zinc content and cell growth between both cells during zinc-deficient culture (data not shown). Thus, the cellular responses to maintain homeostasis of zinc metabolism in response to zinc deficiency appear to function normally in *ZnT1* ^−/−^ *MT* ^−/−^ *ZnT4* ^−/−^ cells, although cytosolic zinc levels markedly increased.

### Extreme zinc supplementation restores TNAP activity in *ZnT1* ^−/−^ *MT* ^−/−^ *ZnT4* ^−/−^ cells

**Figure 5:**
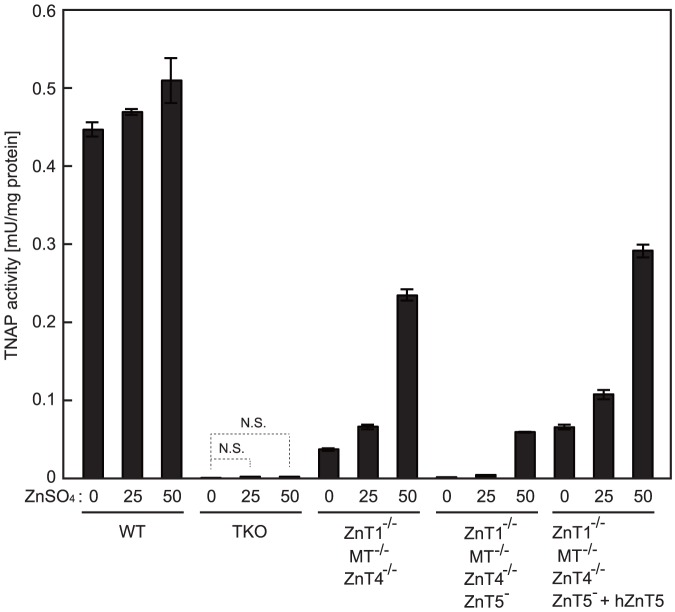
TNAP activity is restored by high zinc supplementation in *ZnT1* ^−/−^ *MT* ^−/−^ *ZnT4* ^−/−^ cells. The indicated cells were cultured in medium supplemented with 0, 25 or 50 µM ZnSO_4_ for 40 h. TNAP activity is the mean ± SD of three independent experiments (N.S., not significant). Note that TNAP activity in *ZnT5* ^−^ *ZnT6* ^−/−^ *ZnT7* ^−/−^ (TKO) cells was never restored.

In the Atox1-ATP7A/ATP7B pathways [13]–[15], intracellular copper increases in Atox1 knockout/knockdown cells do not result in secretory cuproenzyme activation via ATP7A/ATP7B [34], [35], but copper supplementation can recover the activation [36], [37]. Thus, we next examined the effects of zinc supplementation on TNAP activity in *ZnT1* ^−/−^ *MT* ^−/−^ *ZnT4* ^−/−^ cells. The reduced TNAP activity in *ZnT1* ^−/−^ *MT* ^−/−^ *ZnT4* ^−/−^ cells gradually increased in the presence of zinc supplementation of up to 50 µM ZnSO_4_ (Fig. 5), which was the limiting concentration for cells to grow (see Fig. 1A). This is in sharp contrast with that of *ZnT5* ^−^ *ZnT6* ^−/−^ *ZnT7* ^−/−^ (TKO) cells, which could not be restored by zinc supplementation, as shown in a previous study [18]. Zinc supplementation also increased TNAP activity in both *ZnT1* ^−/−^ *MT* ^−/−^ *ZnT4* ^−/−^ *ZnT5* ^−^ cells and *ZnT1* ^−/−^ *MT* ^−/−^ *ZnT4* ^−/−^ *ZnT5* ^−^ cells stably expressing hZnT5. The differences between their activities were caused by the presence or absence of hZnT5 expression, which corresponds to the amount of TNAP activity that is restored via zinc-loading by the ZnT5-ZnT6 hetero-complex in *ZnT1* ^−/−^ *MT* ^−/−^ *ZnT4* ^−/−^ cells. Thus, the zinc transport complexes can supply zinc to TNAP protein in the ESP under conditions where cytosolic zinc levels are extremely increased. These results strongly suggest that ZnT1, MT and ZnT4 play a pivotal role in the activation process of TNAP upstream of the zinc transport complexes.

### Zinc transport activities of ZnT1 and ZnT4 are indispensable for TNAP activation

**Figure 6:**
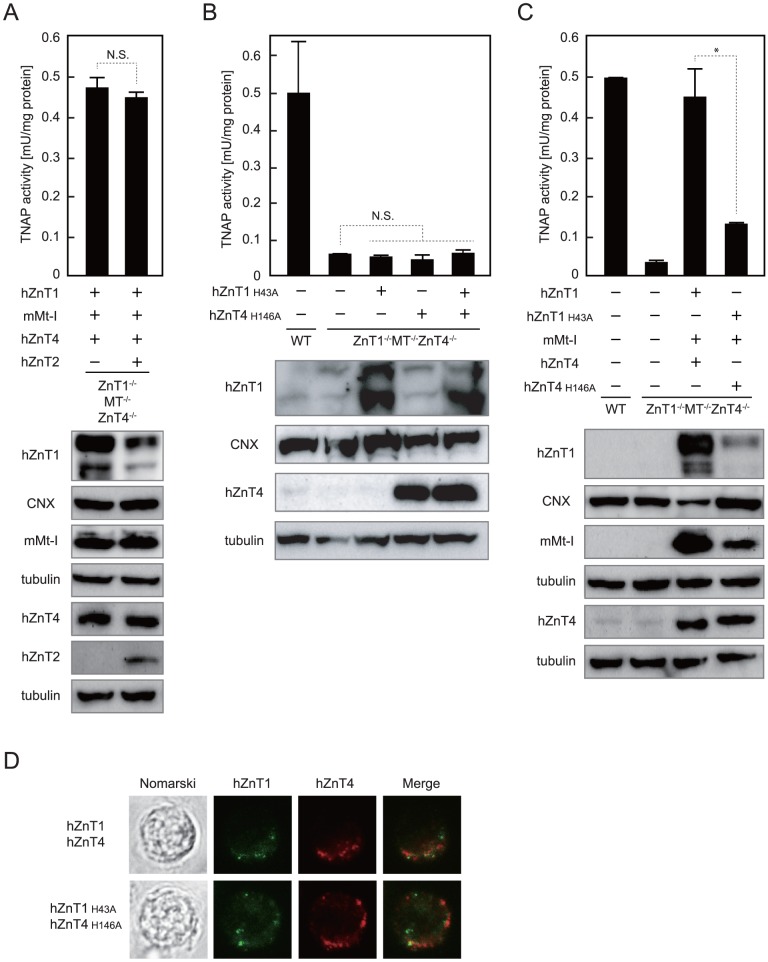
Zinc transport activities of ZnT1 and ZnT4 are required for TNAP activation. (**A**) Expression of hZnT2 did not significantly affect TNAP activity restored by re-expression of all of hZnT1, mMt-I and hZnT4 in *ZnT1* ^−/−^ *MT* ^−/−^ *ZnT4* ^−/−^ cells (*upper panel*). Expression of FLAG-hZnT1, mMt-I, hZnT4-HA and hZnT2-FLAG was confirmed by immunoblot analysis using total cellular or membrane proteins prepared from the indicated cells (*lower panels*). (**B**) Re-expression of zinc transport-incompetent mutants hZnT1 (FLAG-hZnT1 H43A) or/and hZnT4 (hZnT4-HA H146A) failed to significantly restore the reduced TNAP activity in *ZnT1* ^−/−^ *MT* ^−/−^ *ZnT4* ^−/−^ cells (*upper panel*). Expression of FLAG-hZnT1 and hZnT4-HA mutants was confirmed by immunoblot analysis (*lower panels*). (**C**) Expression of hZnT1 and hZnT4 mutants failed to adequately restore reduced TNAP activity, even if mMt-I was expressed (*upper panel*). Expression of FLAG-hZnT1 H43A mutant, mMt-I and hZnT4-HA H146A mutant was confirmed by immunoblot analysis (*lower panels*). In A–C, TNAP activity is expressed as the mean ± SD of three independent experiments (*P<0.01, N.S., not significant, *upper panels*), and tubulin and calnexin (CNX) are shown as loading controls (*lower panels*). (**D**) The subcellular localization of hZnT1, hZnT4 (*upper panels*) and their mutants (*lower panels*) expressed in *ZnT1* ^−/−^ *MT* ^−/−^ *ZnT4* ^−/−^ cells. Nomarski, FLAG-hZnT1 (green), hZnT4-HA (red) and the merged images are shown.

Exogenous expression of hZnT2 did not significantly change reduced TNAP activity in *ZnT1* ^−/−^ *MT* ^−/−^ *ZnT4* ^−/−^ cells, although it reversed cytosolic zinc levels (see Fig. 4A and B). Moreover, hZnT2 expression did not significantly affect TNAP activity restored by re-expression of all counterparts of ZnT1, MT and ZnT4 in *ZnT1* ^−/−^ *MT* ^−/−^ *ZnT4* ^−/−^ cells (Fig. 6A). These results suggest that cooperative zinc handling of ZnT1, MT and ZnT4 is crucial for full activation of TNAP, independently of cytosolic zinc levels. To examine the notion in more depth, we constructed H43A hZnT1 and H146A hZnT4 mutants, in which the essential amino acids for zinc-binding in transmembrane domains are substituted [18], [19], [38]. Loss of zinc transport activity of both mutants was confirmed by the results that re-expression of either mutant failed to reverse the zinc-sensitive phenotype of *ZnT1* ^−/−^ *MT* ^−/−^ *ZnT4* ^−/−^ cells (Table 1). We established *ZnT1* ^−/−^ *MT* ^−/−^ *ZnT4* ^−/−^ cells stably expressing either mutant or both mutants, but the reduced TNAP activity never significantly changed in any of these cases (Fig. 6B). Furthermore, co-expression of both mutants with mMt-I in *ZnT1* ^−/−^ *MT* ^−/−^ *ZnT4* ^−/−^ cells did not result in significant increases in TNAP activity, when compared with that in *ZnT1* ^−/−^ *MT* ^−/−^ *ZnT4* ^−/−^ cells stably re-expressing all of the normal counterparts (Figs. 6C and 2A). Immunofluorescence staining revealed that hZnT1 is dominantly localized to the intracellular compartments/vesicles as shown elsewhere [39], [40], only partially overlapping with hZnT4 in *ZnT1* ^−/−^ *MT* ^−/−^ *ZnT4* ^−/−^ cells, and the mutations of H43A in hZnT1 and H146A in hZnT4 did not significantly change their subcellular localization (Fig. 6D). These results indicate that zinc handling by ZnT1 and ZnT4 in the cytoplasm is required for the activation process of TNAP.

### Homeostasis of the secretory pathway is not significantly impaired in *ZnT1* ^−/−^ *MT* ^−/−^ *ZnT4* ^−/−^ cells

**Figure 7:**
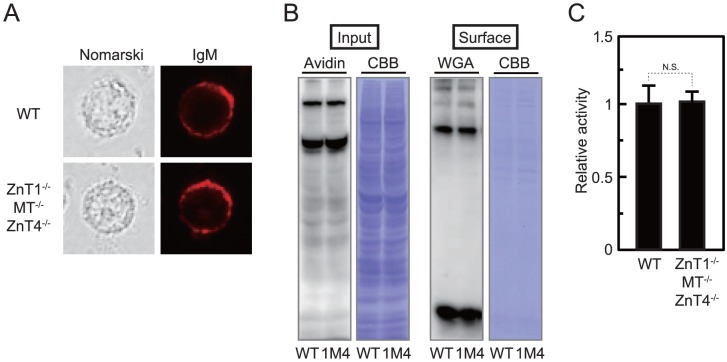
Homeostasis of the secretory pathway is not significantly impaired in ZnT1^−/−^MT^−/−^ZnT4^−/−^ cells. (**A**) Surface IgM expression was not impaired in *ZnT1* ^−/−^ *MT* ^−/−^ *ZnT4* ^−/−^ cells. WT and *ZnT1* ^−/−^ *MT* ^−/−^ *ZnT4* ^−/−^ cells were fixed and immunostained without permeabilization. (**B**) The cell surface proteins biotinylated with membrane-impermeable reagent were almost the same between WT and *ZnT1* ^−/−^ *MT* ^−/−^ *ZnT4* ^−/−^ cells. Surface refers to the solubilized proteins captured using streptavidin beads, while input refers to the aliquot of the biotinylated proteins before avidin capture (that is total cell lysate). In the surface panel, lectin blotting using WGA detects cell surface glycosylated proteins, and CBB staining detects total cell surface proteins. 1M4; *ZnT1* ^−/−^ *MT* ^−/−^ *ZnT4* ^−/−^ cells. (**C**) Secretory *Cypridina* luciferase expression was not significantly different between WT and *ZnT1* ^−/−^ *MT* ^−/−^ *ZnT4* ^−/−^ cells. Both cells transiently transfected with secretory *Cypridina* luciferase reporter were cultured for 4 h after the medium change. Relative activity of *Cypridina* luciferase in the spent medium is shown (the luciferase activity/total cellular proteins in WT cells is defined as 1). Each value is the mean ± SD of three independent experiments (N.S., not significant).

Because the zinc transport complexes cannot fully operate for TNAP activation in *ZnT1* ^−/−^ *MT* ^−/−^ *ZnT4* ^−/−^ cells (see Fig. 3), secretory homeostasis may be disturbed in the cells. Thus, we investigated whether or not loss of ZnT1, MT and ZnT4 impaired homeostasis of the secretory pathway. Because DT40 cells express IgM on the cell surface [41], we first compared its expression in WT and *ZnT1* ^−/−^ *MT* ^−/−^ *ZnT4* ^−/−^ cells. Immunofluorescence staining without permeabilization revealed that expression of IgM was detected on the cell surface in both cells, and that fluorescence intensities were almost the same between both cells (Fig. 7A). The cell surface-localized proteins, which were biotinylated with membrane-impermeable reagent, were also expressed to almost the same levels between both cells (Fig. 7B). Lectin blotting using WGA showed almost no difference between both cells (Fig. 7B), which indicated that glycosylation in the secretory pathway was unaffected in *ZnT1* ^−/−^ *MT* ^−/−^ *ZnT4* ^−/−^ cells. Moreover, additional studies using secretory *Cypridina* luciferase confirmed normal protein secretion in *ZnT1* ^−/−^ *MT* ^−/−^ *ZnT4* ^−/−^ cells. The *Cypridina* luciferase transiently transfected in WT and *ZnT1* ^−/−^ *MT* ^−/−^ *ZnT4* ^−/−^ cells revealed almost the same activities in their spent medium (Fig. 7C). Taken together, homeostasis of the secretory pathway is unlikely to be significantly impaired in ZnT1^−/−^MT^−/−^ZnT4^−/−^ cells, most likely because the residual activities of the zinc transport complexes are enough to maintain it, although they are not enough to fully activate TNAP.

## Discussion

Zinc enzymes are estimated to constitute approximately 3% of total cellular proteins [4], [5], and thought to perform crucial functions in various cellular and physiological processes [1], [5]. However, very little information is available on how zinc is trafficked and transferred to and then coordinated in zinc enzymes and how that is controlled within the cells. The purpose of this study was to investigate how proteins conducting cytoplasmic zinc metabolism relate to TNAP activation in the ESP. We have shown that cooperative functions of ZnT1, MT and ZnT4 are indispensable for full activation of TNAP. Moreover, we found that not cytosolic zinc levels but zinc handling by them is crucial for this process. The zinc transport complexes cannot fully operate to activate TNAP in *ZnT1* ^−/−^ *MT* ^−/−^ *ZnT4* ^−/−^ cells except when extreme zinc is supplemented. These results suggest that the activation process of secretory and membrane-bound zinc enzymes including TNAP are elaborately controlled before zinc is mobilized into the ESP to be supplied to the enzymes. To our knowledge, this is the first report describing molecular evidence of a relationship between proteins conducting cytoplasmic zinc metabolism and activation of secretory or membrane-bound zinc enzymes.

The results of multiple experiments in this study clearly indicate that zinc mobilization functions of ZnT1 and ZnT4 along with MT functions are required for maximal TNAP activation. How do these three proteins contribute to the TNAP activation process? The reduced TNAP activity in *ZnT1* ^−/−^ *MT* ^−/−^ *ZnT4* ^−/−^ cells was almost unchanged by over-expressing the zinc transport complexes (see Fig. 3). Moreover, the reduced activity was not restored when increased cytosolic zinc levels were changed to levels similar to those in WT cells (see Fig. 4). However, extremely high zinc supplementation could restore the reduced activity (see Fig. 5). These results suggest that cooperative functions of ZnT1, MT and ZnT4 may lead to regulation that facilitates cytosolic zinc delivery to the zinc transport complexes to supply zinc into the lumen of the ESP. How zinc is delivered to zinc transporters has been an unsolved question. Therefore, understanding the molecular basis of zinc handling by ZnT1, MT and ZnT4 may provide further clues.

Generally, ZnT1, MT and ZnT4 are thought to regulate cytoplasmic zinc metabolism at different subcellular localizations via different mechanisms; ZnT1 is mainly involved in cytosolic zinc efflux at the plasma membrane, MT is involved in cytosolic zinc chelation, and ZnT4 is involved in cytosolic zinc sequestration in intracellular compartments [6], [8], [15], [42], [43]. Thus, one may ask how they can work together in the TNAP activation process. TNAP activity gradually decreased by disruption of *ZnT1*, *MT* and *ZnT4* genes, and re-expression of them gradually restored this reduction in TNAP activity in a dose–dependent manner. These results suggest that ZnT1, MT and ZnT4 contribute to the process by their own functions and exclude the notion that they are operative only when their functions merge. In our results, immunofluorescence staining reveals the dominant intracellular localization of hZnT1, only partially overlapping with hZnT4, in *ZnT1* ^−/−^ *MT* ^−/−^ *ZnT4* ^−/^cells (see Fig. 6D), which suggests that ZnT1 localized to intracellular compartments/vesicles, not to the plasma membrane, plays a critical role in the TNAP activation process. Considering the direction of zinc transport by ZnT1 and ZnT4, which reversed the zinc-sensitive phenotype of *ZnT1* ^−/−^ *MT* ^−/−^ *ZnT4* ^−/−^ cells in this study (see Table 1), they would transport zinc into the lumen of the intracellular compartments/vesicles, from which zinc would be delivered to the zinc transport complexes located in the ESP. One simple potential model to explain the TNAP activation process is that zinc mobilized by ZnT1 or ZnT4 into the compartments/vesicles is redistributed to the cytosol by ZIP proteins or other zinc transport proteins such as calcium channels, to be supplied to the zinc transport complexes, and that MT would perform supportive functions in this process. Human ZnT2 appears to reside in the different intracellular compartments/vesicles from those where ZnT1 and ZnT4 are localized (Fig. S2), suggesting that specific compartmentalization of zinc by each ZnT transporter may be critical for TNAP activation. The importance of zinc release out of intracellular compartments by ZIP proteins and calcium channels has been described in a number of studies [44]–[50], and a recent report that absence of ZIP13 causes the reduction of TNAP activity may support this notion [51]. The differences of re-expression levels of ZnT1, MT and ZnT4 did not significantly change the restoration rate of TNAP activation (data not shown), which may also support it. Technical difficulty to determine the precise intracellular localization of actual functional zinc transporters is an obstacle to investigating this possibility in more detail. Our strategy using DT40 cells has an advantage in addressing it, because the cells express most of those proteins (ZnTs, MT, ZIPs and calcium channels), which enables us to establish the cells deficient in a combination of multiple genes among them.

This study highlights that TNAP activation is sophisticatedly regulated by specific zinc transport systems including ZnT5, ZnT6, ZnT7 in the ESP, and ZnT1, ZnT4 in the intracellular compartments/vesicles, cooperating with MT. This kind of regulation would be operative in the activation process of a number of zinc enzymes in the ESP, because all of these proteins are ubiquitously expressed [20], [42], [43] and because the strict activation regulation of them is essential to control numerous cellular events [1], [5], [8]. Clarification of TNAP activation mechanism would contribute to determining the function of each protein involved in zinc metabolism, as well as their interplay.

## Supporting Information

Figure S1 **Experimental strategy and targeting constructs.** *A*, Strategy for disruption of all c*ZnT1*, c*MT*, c*ZnT4* and c*ZnT5* genes. Because the c*ZnT5* gene is monosomic in DT40 cells, one targeting construct was used. *B*, Targeted disruption of the c*MT1 and cMT2* genes, which are ∼2 kb apart on chromosome 11. MT1 and MT2 are designed according to (27). Closed boxes indicate exons deduced from the sizes of genomic PCR fragments and the Chicken Genome Resources (http://www.ncbi.nlm.nih.gov/projects/genome/guide/chicken/). The *HisD* or *Bsr* drug resistant marker cassettes were flanked by mutated loxP sites indicated by gray arrowheads. Two targeting constructs, which are designed to disrupt exons encoding the open reading frames of both c*MT* genes, are shown. Gray boxes indicate 5′ and 3′ probes used for confirming disruption of the c*MT* genes. Southern blot analysis confirmed homologous recombination at c*MT* loci (*below left*). Genomic DNA prepared from the indicated genotypes (wild-type shown as WT, *MT* ^+/−^ and *MT* ^−/−^) was digested with *Eco*RI or *Bam*HI, and hybridized with the 5′ or 3′ probes shown above. RT-PCR analysis indicated the absence of both c*MT1* and c*MT2* mRNA expression in *MT* ^−/−^ cells (*below right*). Southern blot and RT-PCR were performed as described previously (23) and in the main text.
(EPS)

Figure S2 **Human ZnT2 expressed in** ***ZnT1*** **^−/−^** ***MT*** **^−/−^** ***ZnT4*** **^−/−^ cells is localized to different intracellular compartments/vesicles from those where hZnT1 and hZnT4 are located.** FLAG-hZnT1and hZnT2-HA and the merged image (*upper panels*), and hZnT2-FLAG and hZnT4-HA and the merged image (*lower panels*), are shown.
(EPS)

Table S1 **Primers used for RT-PCR analysis.**
(DOC)
