# Supplementary material for: Cooperative Functions of ZnT1, Metallothionein and ZnT4 in the Cytoplasm Are Required for Full Activation of TNAP in the Early Secretory Pathway
Source: PLoS One. 2013 Oct 18;8(10):e77445. doi: 10.1371/journal.pone.0077445 (PMC3799634; doi:10.1371/journal.pone.0077445)
Supplement: Table S1 — Primers used for RT-PCR analysis. (DOC) [file pone.0077445.s003.doc]

**Table S1. Primers used for RT-PCR analysis**

Gene Forward (5’ to 3’) Reverse (5’ to 3’) Length (bp) cycles

cZnT1 CGGCTGCTGTGCATGCTGGCGCTCACCTTC TGGGAACAGTCTGTAGAAGTATGAGGGCCG 1010 34

cZnT4 ATGGCCGGGCCCGGCCTGTGGAGCAGCATC AGGCACTGGAACTCTGACAACTTGCGCAGG 1288 34

cZnT5 GCTGTTTGAACACAGTGATGTGGTTGTGCT AGTGCCTTCAGGAGAATAGCCAATAAGGGT 778 28

cZnT6 TTCCGGTGCGGGGAGTACTATGGGGACAAT CGGACATGGACCGATCCAGCCAAAGTGCCA 905 34

cZnT7 ATGCTGCCCCTCTCCATTAAGGACGATGAG CTACATGGCTGCAACATCAATTTGTATGTA 1137 34

cMT1 ATGGACTCCCAGGACTGCCCTTGTGCCACC CTACTTGCAGCAGCTGCACTTGGCGGAGGG 192 40

cMT1* GCGGCACCTGCACGTGTGGAGACAACTGCA GCCCTTTGCAGACGCAGCCCTGTGCACACT 129 21

cMT2 ATGGACCCTCAGGACTGCACTTGTGCTGCT TCAGTGGCAGCAGCTGCACTTGCTGCTGGC 192 40

cMT2* TGACTCCTGCTCCTGTGCTGGGTCGTGCAA GGTTCCTTGCAGACACAGCCCTTGGCACAG 129 21

cTNAP CGCGGATCCATGAAGGCTTTCCTCCTCACC CTAGGAGCTGCAGTGCGCACGGTTGGGGCC 1506 40

cβactin TGCGTGACATCAAGGAGAAGCTGTGCTACG GAAGCATTTGCGGTGGACAATGGAGGGTCC 500 40

* Used for nested PCR. The annealing temperature was 68 °C for all reactions.
